# Prevalence of depression and its associated factors among undergraduate admission candidates in Bangladesh: A nation-wide cross-sectional study

**DOI:** 10.1371/journal.pone.0295143

**Published:** 2023-11-30

**Authors:** Md Abu Bakkar Siddik, Md Nafiul Hasan, Al Mahmud, Morioum Sarkar Munmun, Mahmudul Hasan Milad, Akher Ali, Zobayer Ahmed, Md Jamal Uddin

**Affiliations:** 1 Department of Forestry Engineering, Nanjing Forestry University, Nanjing, China; 2 Department of Statistics, Shahjalal University of Science & Technology, Sylhet, Bangladesh; 3 Department of Microbiology, University of Dhaka, Dhaka, Bangladesh; 4 Department of Public Health, Birmingham City University, Birmingham, United Kingdom; 5 Department of Statistics, Jahangirnagar University, Dhaka, Bangladesh; 6 Department of Economics & Banking, International Islamic University Chittagong, Chittagong, Bangladesh; 7 Faculty of Graduate Studies, Daffodil International University, Dhaka, Bangladesh; Hamdard University Bangladesh, BANGLADESH

## Abstract

**Background:**

The undergraduate admission test is one of the most stressful assessments in a student’s life, as it is required for admission to any of Bangladesh’s public universities or medical colleges. Those taking the admissions test are under a lot of pressure to perform well. This study aimed to determine the prevalence of clinical depression among Bangladeshi admission candidates and the factors that contribute to it.

**Methodology:**

Patient Health Questionnaire (PHQ-9) and other socio-demographic information were collected from 5263 students from all over Bangladesh. Apart from descriptive statistics and chi-square tests, an ordinal logistic regression model was also applied to determine the factors associated with depression.

**Results:**

The study revealed that among the undergraduate admission applicants, 74% of individuals were affected by depression, while 26% experienced moderate depression, 26% experienced moderately severe depression, and 22% experienced severe depression. The level of depression among females was 1.8 times higher than the male admission candidates. Our analyses found that gender (p <0.001), exercise (p <0.001), pre-marital relationships (p <0.001), daily study time less than 3 hours (p <0.001), practice of religion (p <0.001), victim of blackmail (p <0.001), family unrest (p <0.001), major illness (p <0.001), COVID-19 infection (p <0.001), GPA in higher secondary (p <0.001), mental problem (p <0.001), all categories of the variable confidence level for exam preparation (p <0.001) had a significant impact on increasing depression.

**Conclusion:**

The research found a severe rate of depression among Bangladeshi undergraduate admission candidates. Interactive mental health care programs must include family and teachers to tackle the problem. To alleviate mental stress and depression, students should learn to nurture their mental health.

## Introduction

There is a growing body of research that indicates an increasing incidence of mental health disorders among students, particularly those attending secondary schools and institutions of higher education [1]. The transition from high school to university entails a multitude of transformations across various domains, encompassing social, educational, and emotional aspects of development [2]. In numerous nations, such as Bangladesh, India, Germany, Russia, and France, it is customary for individuals who have completed their secondary education to undergo an entrance examination to secure admission into higher education institutions, and acquisition of a university degree is widely regarded as a noteworthy achievement, as it provides individuals with novel frameworks and proficiencies, enabling them to delve into an extensive reservoir of knowledge [3].

The undergraduate admission examination is one of the most stressful parts of a student’s life because this test is the first step toward achieving a career goal, particularly in Bangladesh. Due to the restricted number of available seats, even those students who are eligible to apply and take the admissions exam are not guaranteed admission to the institution [3]. Students must overcome the obstacle of the admissions exams and pass the exam to secure their places. For example, in the academic year 2021–22, about 1.4 million students throughout the nation participated in high school and equivalent examinations, with 189,169 achieving the highest grade of 5 [4]. Bangladesh has a total of 51 public universities and 56 public medical and dental colleges. Approximately 60,000 places are available at universities, while another 5,000 are available at medical and dental colleges [5]. These limited seats cause enormous psychological pressure on the admission candidates [6]. The majority of applicants fail it, and consequently, they are denied admission. Many of them even commit suicide for not getting admission to the institutions [7,8]. Most of the students participating in the admission test are adolescents.

It had been reported by researchers that the prevalence rate of depression among adolescents in India are 25.8 percent [9]. Iran had a rate of 52.6 percent [10], which is much higher than Pakistan’s 17.2 percent [11], which stands in comparison to Malaysia’s 26.2 percent [12] and Sri Lanka’s 36 percent [13], 33 percent in China [14], and around 40 percent in South Korea [15]. Moreover, among adolescents, depression was associated with anxiety, GPA, major physical illness, smoking, drug or alcohol, decreasing academic excellence, self-injurious behaviors, and suicidal thought [16–18].

Several researchers have studied the mental condition of the undergrad (either university or medical colleges) admission candidates. The level of depression is associated with gender, result grade, family income, study time, and social activities [19,20]. High self-reported symptoms of depression reflect exam-related stress, expectations from society, worrying about future performance connected to university placement uncertainties, etc [21].

Pandemic caused by the Covid-19 virus has had a profound influence on both physical and mental health, as has been thoroughly documented by researchers from all around the world, Bangladesh was not an exception [22]. The mental well-being of adolescents was significantly impacted by the pandemic, leading to the emergence of depression and various other mental health conditions [23]. Due to the miserable conditions caused by the pandemic, it became necessary to reschedule the university entrance exams [24].

All the previous studies that have been conducted on depression in Bangladesh are too specified, either school-college or undergraduate medical-university students. A few studies have covered one of the most crucial periods for adolescents after passing college, the undergraduate admission time, but the researchers hardly found any study conducted to assess the significant factors as well as covid-19. This study aims to fill this study gap. Hence, the main objective of the study was to assess the prevalence of depression among undergraduate admission candidates in Bangladesh. The study also aimed to identify the impact of several socio-demographic factors, including covid-19 on the current mental health of undergraduate admission candidates in Bangladesh.

## Methodology

### Study design, study settings, data collection procedure and sample size

A cross-sectional study was conducted in all administrative divisions of Bangladesh from AprilJune 2022 through JulyOctober 2022. The data was collected using stratified cluster sampling technique. The administrative divisions were considered as stratum where the various coaching institutes within the divisions were taken as clusters. Our respondents were students who attained undergraduate admission tests for the academic year 2021–2022. Data was collected online using Google Forms. The data enumerator visited the institutes and distributed via institutional groups in social media (Telegram, WhatsApp, Messenger, and Facebook) comprising of the students at the institute. In the questionnaire, socio-demographic and Patient Health Questionnaire (PHQ-9) items were included. The PHQ-9 questions were valid and reliable for this data as Cronbach’s alpha value of 0.78. To establish the practicability and efficacy of the research, a pilot study consisting of fifty-one students was carried out. After that, they were disregarded in the further examination of the data. The questionnaire passed through a few rounds of edits before the final research was ever carried out to make sure it met all of the requirements.

The Sample size (n) was calculated as follows:

$$\text{Sample}\;\text{Size}=\frac{\frac{z^{2}\cdot p(1-P)}{e^{2}}}{1+\frac{z^{2}\cdot p(1-p)}{e^{2}N}}=4211$$

If 20% non-response (80% response) then the final sample size will be: n = 4211/0.80 = 5263

Where,

N = 1.4 million; the total number of students who attained undergraduate admission test

Z = 1.96, Z value for 95% confidence limits,

P = Assumed prevalence (i.e., 0.50 for 50%)

e = Desired precision (e.g., 0.02).

### Ethical approval

Biostatistics, epidemiology, and public health research group, Department of Statistics, Shahjalal University of Science and Technology, Sylhet-3114, Bangladesh, has approved the ethics application **(Ref: PR-003)**. Ethical guidelines established by the institution’s or country’s research committee, as well as the 1964 Helsinki Declaration and the later amendments of it or other equivalent ethical norms, guided every step of the process. Those who ensured written consent to participate in the study were included in this study.

### Inclusion and exclusion criteria

The inclusion criteria used in this research were as follows: i) Individuals seeking admission to a university who have completed the Higher Secondary Certificate (HSC) test during the 2021–22 academic session. ii) Individuals applying for admission to a university who possess Bangladeshi nationality. The exclusion criteria used in this investigation were as follows: i) Students enrolled in educational institutions that use English as the language of instruction; ii) Students in Vocational curriculums, iii) Students those studied abroad In Bangladeshi curriculum.

### Socio-demographic variable

Age, district name, marital status (married, unmarried), gender (male, female), family economic condition (lower class, lower middle class, middle class, middle-upper class, upper class), uses of social media (yes, no), religion (Islam, Hinduism, Others), any types of addiction (yes, no), pre-marital affair (yes, no), sexual experience (yes, no), regular physical activities (yes, no), duration of the study (below three hours, three to four hours, four to five hours, five to six hours, above six hours), religiosity (yes, no), smoking (yes, no), a victim of any types of blackmail (yes, no), family unrest (yes, no), major physical illness (yes, no), affected by covid-19 (yes, no), GPA (GPA-3, GPA-4, GPA-5), exam preparation status (not confident, less confident, confident, more confident), family mental illness history (yes, no) were considered as socio-demographic variables.

### Outcome variables

The Patient Health Questionnaire was used to gauge the degree of depression experienced by the respondent (PHQ-9). When it comes to diagnosing depression in a person, the PHQ-9 is one of the most well-known and easy-to-use questionnaires. For PHQ-9 questions, the 4-point Likert scale ranges from “0 = not at all” to “3 = almost every day”. Based on scores ranging from 0 to 4, 5 to 9, 10 to 14, 15 to 19, and 20 to 27, respectively, the severity of depression was assigned to one of five groups: None-minimal, mild, moderate, moderately severe, and severe. Whereas, score more than 10 was counted as depressed category [25]. **S1 Appendix** is a list of all the questions and scales used in the PHQ-9 questionnaires.

### Statistical analysis

The descriptive analysis was used to summarize the socio-demographic and depression measuring variables. Respondents were categorized into various levels of depression by measuring score bands of PHQ-9 Likert scale. Pearson Chi-square test was used to assess the association between different variables with depression. An ordinal logistic regression model was fitted between depression categories as dependent variable and all the other variables as independent variables. Odds ratios (OR) and 95% confidence intervals for the OR were derived for all the categorical variables. The distributions of depression according to “gender”, “use of social media”, “ever blackmailed”, and “having family problems” were presented in bar charts. A Pie chart presents the percentage of admission students suffering from various levels of depression.

A Multivariable adjusted ordinal logistic regression model was fitted to this data to assess the relationship between various explanatory variables and the dependent variable which was the depression level. We used an ordinal logistic regression model as the response for depression levels which are in a natural order. Among various types of ordinal logistic regression models, we decided to use the Proportional Odds model which seeks to forecast the likelihood that a case will fall within or exceed a certain level of a response variable, which is the complementary direction from one or more explanatory variables. Various computer programs parameterize this model as the function given below:

$$ln\left(\frac{\mathrm{Pr}\;(y\leq j)}{\mathrm{Pr}\;(y>j)}\right)=ln\;(odds\;(y\leq j))=\theta_{j}‐\left(\beta_{1}X_{1}+\cdots +\beta_{k}X_{k}\right)$$

The function above resulted in a more consistent reading with how one would generally interpret the trend of effects in the framework of linear regression. where Pr (*y* ≤ *j*) is denoted as the cumulative probability of an event (*y* ≤ *j*); *θ*_*j*_ is the respective constant term/intercepts; and *β*_*k*_ are the of regression coefficients that corresponds to the *X*_*k*_ covariates.

Assuming that categories on the dependent variable were ordered ascendingly, that was, moving from lower to higher values on the dependent variable, we could interpret an estimated regression slope as the predicted change in the log odds or logits of a case falling above a given category j on the dependent variable while holding the other predictors constant.

Higher scores on X are related with a larger likelihood that a case would fall into group j, as compared to lower category. If a negative slope (*β*_*k*_) is inserted into the preceding equation, the result is (*β*_*k*_), which indicates that higher X scores are linked with a larger risk of dropping at or below group j and a lower probability of being in a higher category j. Lastly, each threshold represented the cumulative log odds of an instance falling into group j or below, when the predictors were zero.

## Results

### Demographic variables

**Fig 1:**
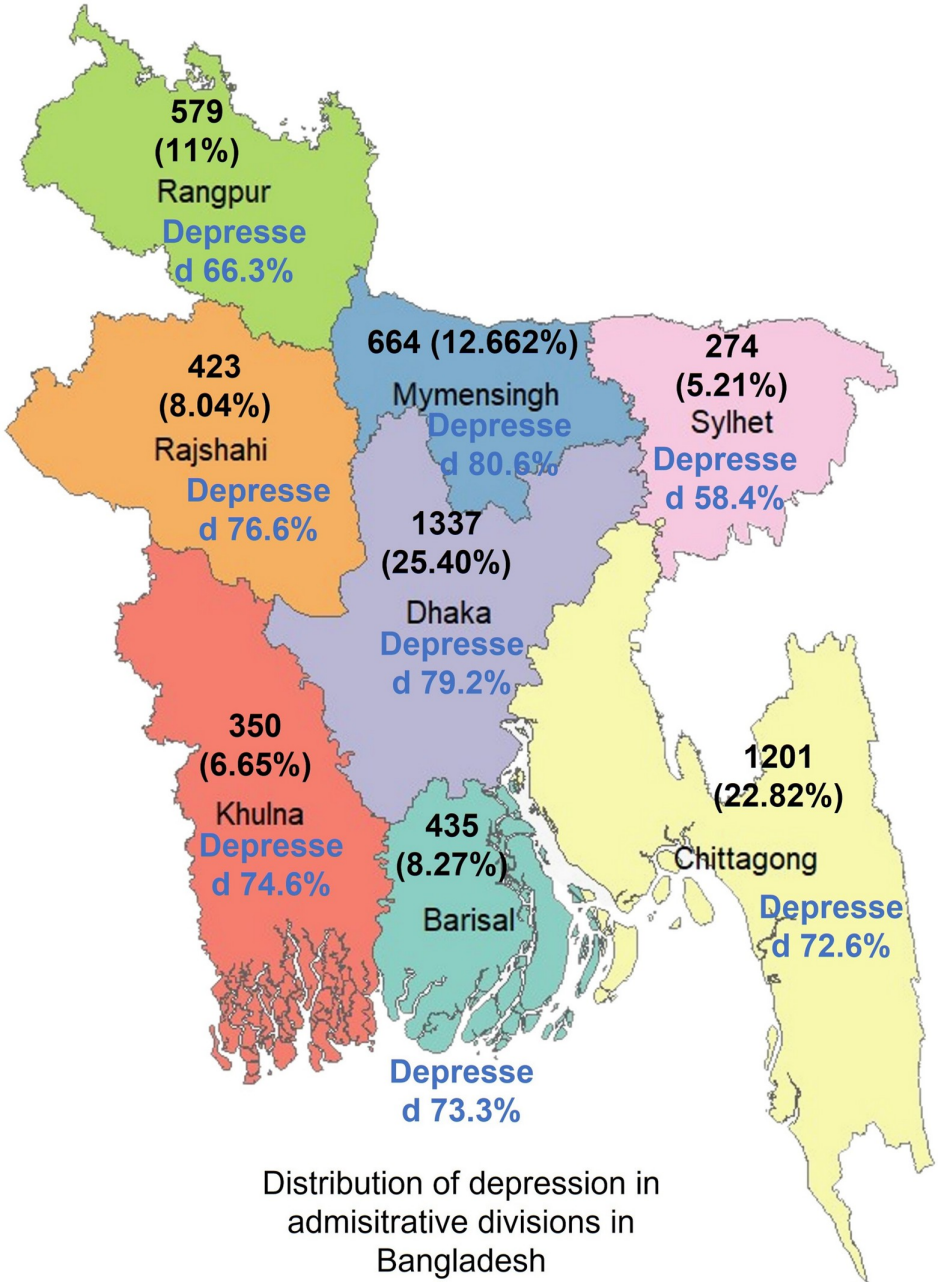


**Table 1** showed the demographic of this research. A total of 5263 respondents were included in the survey. Among the participants, 62.3% were female and 37.7% were male, 6.8% believe in Hinduism, 92.9% were Muslim and 0.3 believed in others. 97.8% were unmarried and the rest were married. The mean age of the respondents was 19.31 (SD = 1.114) years, whereas the age range of the respondents was 16–24 years. Additionally, **Fig 1** illustrated the geographic distribution of respondents across 8 administrative divisions of Bangladesh. It indicated that the Dhaka division had the highest number of respondents at 25.40%, followed by Chittagong with 22.82%. On the other hand, Sylhet had the lowest number of respondents at 5.21%. In this study, we did not consider ’division’ as a factor to assess depression.

**Table 1:** Socio-demographical variable’s distribution by different level of depression.

Variables	Levels	None Minimal Depression	Mild Depression	Moderate Depression	Moderately Severe Depression	Severe Depression	P-Value
		Count (%)	Count (%)	Count (%)	Count (%)	Count (%)	
Age	Mean age 19.31, SD = 1.114						
**Gender**	Female	166 (3.2)	491 (9.3)	799 (15.2)	951 (18.1)	873 (16.6)	<0.001
	Male	212 (4)	480 (9.1)	573 (10.9)	436 (8.3)	282 (5.4)	
**Family Income**	Lower-middle class	67 (1.3)	168 (3.2)	249 (4.7)	254 (4.8)	172 (3.3)	<0.001
	Middle class	77 (1.5)	227 (4.3)	315 (6)	300 (5.7)	211 (4)	
	Middle-upper class	86 (1.6)	199 (3.8)	329 (6.3)	331 (6.3)	303 (5.8)	
	Upper class	65 (1.2)	187 (3.6)	249 (4.7)	279 (5.3)	279 (5.3)	
	Lower class	83 (1.6)	190 (3.6)	230 (4.4)	223 (4.2)	190 (3.6)	
**Use of social media**	No	45 (0.9)	54 (1)	81 (1.5)	79 (1.5)	83 (1.6)	<0.001
	Yes	333 (6.3)	917 (17.4)	1291 (24.5)	1308 (24.9)	1072 (20.4)	
**Religion**	Hinduism	40 (0.8)	65 (1.2)	90 (1.7)	93 (1.8)	69 (1.3)	0.11
	Islam	337 (6.4)	904 (17.2)	1278 (24.3)	1290 (24.5)	1079 (20.5)	
	Other	1 (0)	2 (0)	4 (0.1)	4 (0.1)	7 (0.1)	
**Exercise**	No	267 (5.1)	783 (14.9)	1178 (22.4)	1256 (23.9)	1096 (20.8)	<0.001
	Yes	111 (2.1)	188 (3.6)	194 (3.7)	131 (2.5)	59 (1.1)	
**Pre-marital relationship**	No	328 (6.2)	802 (15.2)	1108 (21.1)	1029 (19.6)	837 (15.9)	<0.001
	Yes	50 (1)	169 (3.2)	264 (5)	358 (6.8)	318 (6)	
**Study time**	3 to 4 hours	40 (0.8)	123 (2.3)	188 (3.6)	187 (3.6)	132 (2.5)	<0.001
	4 to 5 hours	41 (0.8)	121 (2.3)	204 (3.9)	211 (4)	134 (2.5)	
	5 to 6 hours	64 (1.2)	155 (2.9)	241 (4.6)	237 (4.5)	156 (3)	
	Below 3 hours	39 (0.7)	131 (2.5)	224 (4.3)	306 (5.8)	371 (7)	
	More than 6 hours	194 (3.7)	441 (8.4)	515 (9.8)	446 (8.5)	362 (6.9)	
**Practice religion**	No	21 (0.4)	67 (1.3)	116 (2.2)	179 (3.4)	196 (3.7)	<0.001
	Yes	357 (6.8)	904 (17.2)	1256 (23.9)	1208 (23)	959 (18.2)	
**Smoking**	No	362 (6.9)	925 (17.6)	1321 (25.1)	1330 (25.3)	1111 (21.1)	0.78
	Yes	16 (0.3)	46 (0.9)	51 (1)	57 (1.1)	44 (0.8)	
**Experienced any type of blackmailing recently**	No	370 (7)	931 (17.7)	1283 (24.4)	1246 (23.7)	961 (18.3)	<0.001
	Yes	8 (0.2)	40 (0.8)	89 (1.7)	141 (2.7)	194 (3.7)	
**Family problem**	No	331 (6.3)	736 (14)	851 (16.2)	640 (12.2)	366 (7)	<0.001
	Yes	47 (0.9)	235 (4.5)	521 (9.9)	747 (14.2)	789 (15)	
**Sickness**	No	361 (6.9)	895 (17)	1225 (23.3)	1187 (22.6)	901 (17.1)	<0.001
	Yes	17 (0.3)	76 (1.4)	147 (2.8)	200 (3.8)	254 (4.8)	
**Addressed COVID-19 infection**	No	319 (6.1)	760 (14.4)	1073 (20.4)	1021 (19.4)	795 (15.1)	<0.001
	Yes	59 (1.1)	211 (4)	299 (5.7)	366 (7)	360 (6.8)	
**Mental problem**	No	359 (6.8)	918 (17.4)	1259 (23.9)	1240 (23.6)	978 (18.6)	<0.001
	Yes	19 (0.4)	53 (1)	113 (2.1)	147 (2.8)	177 (3.4)	
**Exam Preparation**	Confident	188 (3.6)	501 (9.5)	764 (14.5)	754 (14.3)	596 (11.3)	<0.001
	Less More confident	88 (1.7)	277 (5.3)	388 (7.4)	386 (7.3)	255 (4.8)	
	More confident	91 (1.7)	154 (2.9)	126 (2.4)	73 (1.4)	53 (1)	
	Not confident	11 (0.2)	39 (0.7)	94 (1.8)	174 (3.3)	251 (4.8)	
**Sexual experience**	No	347 (6.6)	885 (16.8)	1220 (23.2)	1246 (23.7)	979 (18.6)	<0.001
	Yes	31 (0.6)	86 (1.6)	152 (2.9)	141 (2.7)	176 (3.3)	
**Marital status**	Married	9 (0.2)	23 (0.4)	29 (0.6)	29 (0.6)	26 (0.5)	0.99
	Unmarried	369 (7)	948 (18)	1343 (25.5)	1358 (25.8)	1129 (21.5)	
**SSC GPA**	GPA-5	158 (3)	470 (8.9)	731 (13.9)	852 (16.2)	804 (15.3)	<0.001
	Not GPA-5	220 (4.2)	501 (9.5)	641 (12.2)	535 (10.2)	351 (6.7)	

### Prevalence of depression

**Fig 2:**
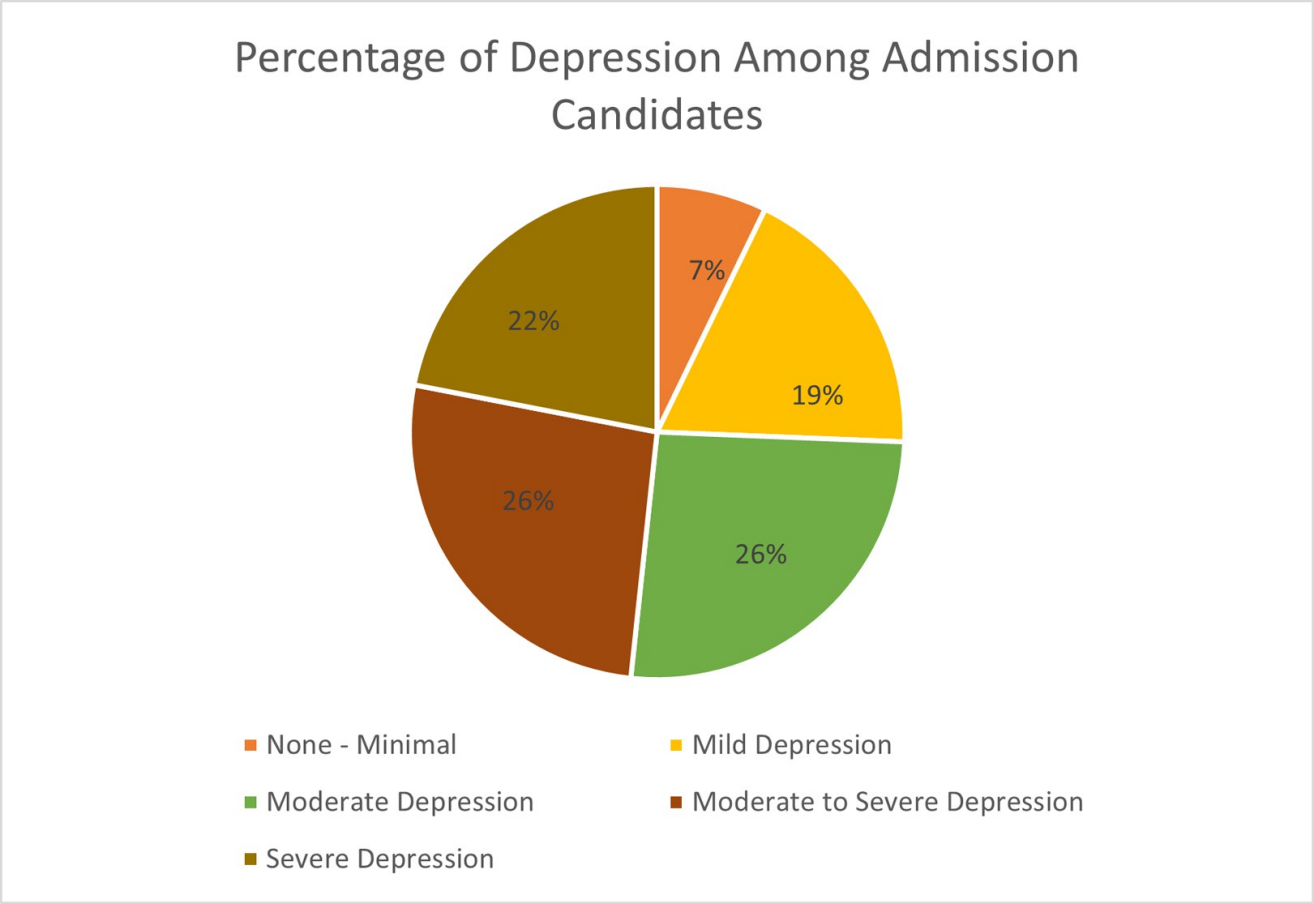


**Fig 2** outlined the prevalence of depression among the participants. We found 74% suffered from depression, while 26% were from moderate, 26% moderately severe, and 22% were suffering from severe levels of depression.

**Fig 3:**
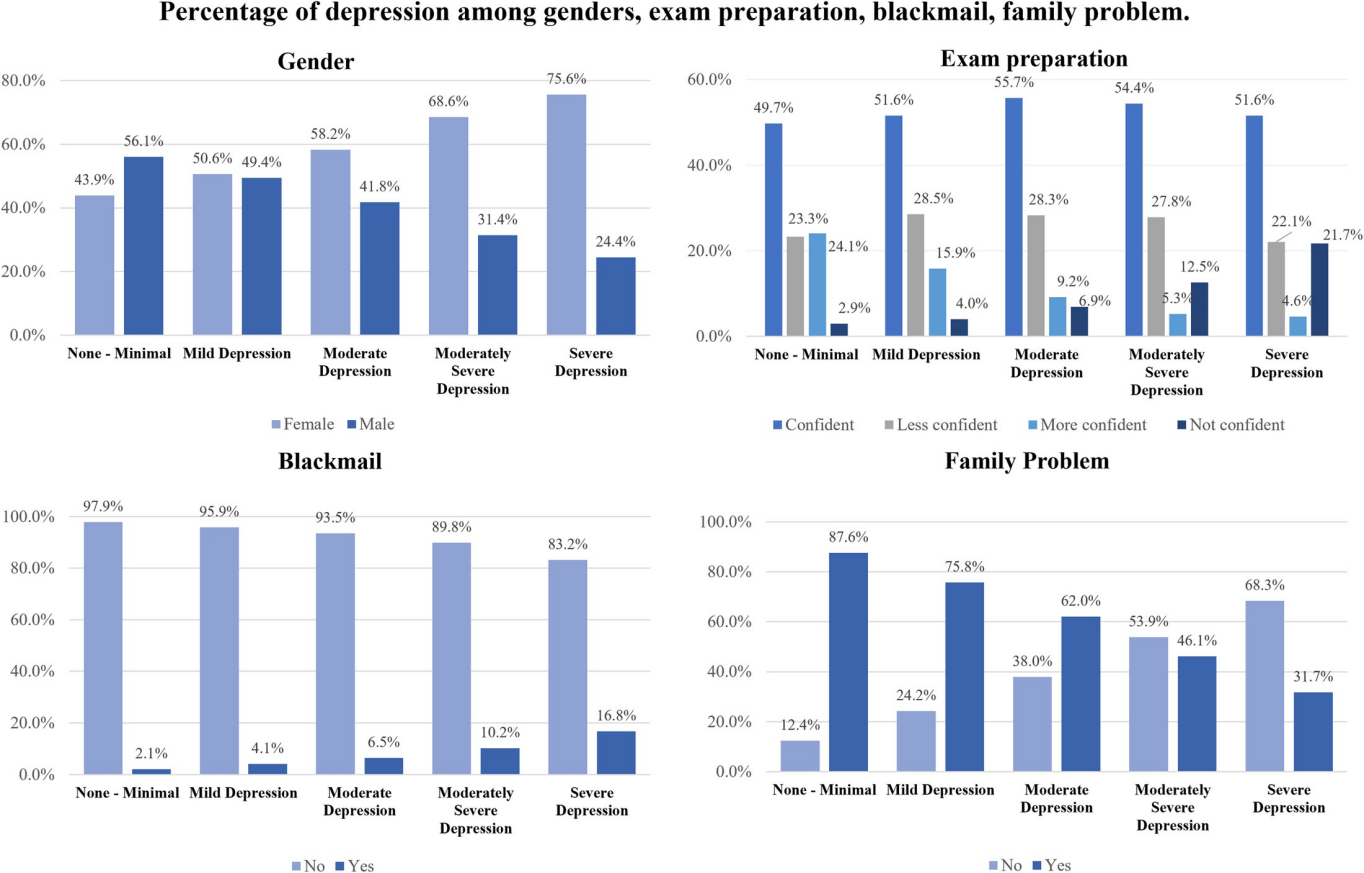


**Fig 3** showed the distribution of depression levels according to genders, exam preparation, blackmail (recently experienced or not) and family problems (whether there is unrest in the family). The opposite trend of depression between males and females, not confident & more confident, blackmailed or not and having a family problem & not having a family problem were very apparent in the bar chart.

From **Table 1** we found that 49.9% of female and 24.6% of male students were depressed. Among the females, 15.2% suffered from moderate depression, 18.1% from moderately severe, and 16.6% suffered a from severe level of depression. Whereas 10.9%, 8.3%, and 5.4% were suffering from moderate, moderately severe, and levels of depression, respectively (p <0.001). Family income (p <0.001), use of social media (p <0.001), exercise (p <0.001), pre-marital relationships (p <0.001), practice of religion (p <0.001), victim of Blackmail (p <0.001), family unrest (p <0.001), major illness (p <0.001), all categories of exam preparation (p <0.001), GPA (p <0.001) had significant effect on increasing depression among the undergrad admission candidates of Bangladesh.

### Factors associated with depression

**Table 2** portraited the estimates, log odds and P-values of the fitted ordinal logistic model. The dependent variable had 5 depression levels as None or minimal, Mild, Moderate, Moderate to severe, and severe. The levels of depression represented lower to higher levels of depression among the respondents for which we fitted the ordinal logistic regression model to predict the relationship between depression and various explanatory variables.

**Table 2:** Estimates of multivariable adjusted ordinal logistic regression model.

VARIABLES	LEVELS	ESTIMATES	OR (95% CI)	P- Value
**Age**		-0.01	0.985 (0.936–1.035)	0.53
**Gender**	Female	0.58	1.79 (1.595–1.981)	<0.001
	Male	0	1	
**Exercise**	No	0.67	1.946 (1.681–2.273)	<0.001
	Yes	0	1	
**In a pre-marital Relationship**	Yes	0.21	1.23 (1.09–1.391)	<0.001
	No	0	1	
**Daily study time**	3 to 4 hours	0.04	1.036 (0.882–1.218)	0.66
	4 to 5 hours	0.07	1.074 (0.917–1.256)	0.38
	5 to 6 hours	0.09	1.093 (0.946–1.267)	0.22
	Below 3 hours	0.33	1.393 (1.205–1.626)	<0.001
	More than 6 hours	0	1	
**Practice religion**	No	0.34	1.402 (1.203–1.677)	<0.001
	Yes	0	1	
**Experienced any type of blackmailing recently**	Yes	0.68	1.972 (1.653–2.384)	<0.001
	No	0	1	
**Having family problem**	Yes	1.06	2.885 (2.597–3.216)	<0.001
	No	0	1	
**Suffering from any kind of major sickness**	Yes	0.58	1.786 (1.557–2.107)	<0.001
	No	0	1	
**Covid infected**	Yes	0.22	1.24 (1.11–1.40)	<0.001
	No	0	1	
**GPA in HSC**	GPA5	0.40	1.48 (1.32–1.67)	<0.001
	Not GPA5	0	1	
**Suffering from any kind of mental problem**	Yes	0.23	1.25 (1.06–1.50)	<0.001
	No	0	1	
**Confidence level for exam preparation**	Confident	-0.63	0.53 (0.44–0.64)	<0.001
	Less confident	-0.79	0.46 (0.38–0.55)	<0.001
	More confident	-1.43	0.24 (0.19–0.3)	<0.001
	Not confident	0	1	

As the model predicted cumulative odds for an instance to be in higher levels as opposed to the lower levels of the dependent variable for changing from reference to other categories of explanatory variables, we observed from the table that, the cumulative odds for female admission candidates to be in higher levels of depression than lower levels of depression are approximately 1.8 times (95% CI (1.595–1.981) higher than that of male candidates.

Candidates who did not practice physical exercise were almost 2 times (95% CI (1.681–2.273), P-value <0.001) more likely to be in higher depression levels as opposed to the candidates who do physical exercise. In addition, those are engaged in pre-marital relationships had 1.2 times (95% CI (1.09–1.391), P-value <0.001) higher odds of being in higher levels of depression as opposed to lower levels than the candidates who did not have pre-marital relationships.

In various study durations only one level was found to be statistically significant, candidates who study less than 3 hours were 1.4 times (95% CI (1.205–1.626), P-value <0.001) more likely to be in higher depression levels than the candidates who had a study time of more than 6 hours. The cumulative odds of participants being in higher levels of depression who were less practicing religion is 1.4 times (95% CI (1.203–1.677), P-value <0.001) higher than those practicing ones.

The cumulative odds of being in higher levels of depression as opposed to lower levels of depression increased by almost 2 times, (95% CI (1.653–2.384), P-value <0.001) for students who had experienced blackmailing recently, and almost 3 times for candidates that were suffering from family problems. Similarly, for applicants who had a major sickness, affected by COVID-19, or had mental problems, the probabilities of being in greater levels of depression as compared to lower levels vary by cumulative factors of 1.8 times (95% CI (1.557–2.107), P-value <0.001)) and 1.25 times (95% CI (1.06–1.50), P-value <0.001), respectively.

Applicants who were more confident about the exam had fewer odds of being in higher levels of depression than less confident candidates. In the log odds scale by taking the reciprocal of the tabulated odds we found that the cumulative odds that candidates who were “Not confident” with the exam preparation are 4 (1/0.53) times more likely to be in higher depression levels than “More confident” candidates. Applicants with higher GPA in HSC had higher cumulative odds of being in higher depression levels by a factor of 1.4 times (95% CI (1.32–1.67), P-value <0.001). Age was statistically insignificant based on the data set.

The most important assumption for ordinal logistic regression was the proportional odds assumption. Which indicated whether the effects of the explanatory variables on the cumulative likelihood of moving up a category on the dependent variable were constant across categories, the test of this assumption came insignificant at a 95% level of significance with p-value = 0.082 (Chi squared 68.986, df 54). Which successfully met the assumption of the model.

### Stratified analysis based on gender

The data set was split into two parts based on the gender of the respondents, and the ordinal logistic regression model is fitted. In a test of parallel lines only the models for male respondents had fulfilled the assumption at a 5% level of significance (Male, Chi-square 42.194, P-value 0.805), and for female’ model did not (Female, Chi square 89.012, p-value 0.001). Among the variables that were both significant among both genders, the odds ratios showed a similar trend of depression which was demonstrated in **Table 3**. Only the variable about mental problems came insignificant in the male’s model (OR = 1.18, 95% CI (0.630–1.126), P-value = 0.24).

**Table 3:** Stratified analysis based on gender.

VARIABLES	LEVELS	MALE		FEMALE	
		OR (95% CI)	P-value	OR (95% CI)	P-value
**Age**	Mean age 19	1.02 (0.951–1.106)	0.5	0.96 (0.89–1.02)	0.19
**Exercise**	No	1.98 (1.623–2.416)	<0.001	1.95 (1.54–2.46)	<0.001
	Yes	1		1	
**In a pre-marital Relationship**	Yes	1.28 (1.051–1.572)	0.01	1.2 (1.03–1.4)	0.02
	No	1		1	
**Daily study time**	3 to 4 hours	1.01 (0.781–1.308)	0.93	1.04 (0.85–1.29)	0.68
	4 to 5 hours	1.04 (0.803–1.351)	0.75	1.1 (0.9–1.34)	0.36
	5 to 6 hours	1.08 (0.852–1.376)	0.51	1.1 (0.91–1.32)	0.32
	Below 3 hours	1.36 (1.071–1.731)	<0.001	1.41 (1.16–1.71)	<0.001
	More than 6 hours	1		1	
**Practice religion**	No	1.29 (1.021–1.633)	0.03	1.51 (1.2–1.92)	<0.001
	Yes	1		1	
**Experienced any type of blackmailing recently**	Yes	2.31 (1.699–3.155)	<0.001	1.81 (1.44–2.27)	<0.001
	No	1		1	
**Having family prob**	Yes	2.61 (2.193–3.109)	<0.001	3.07 (2.68–3.52)	<0.001
	No	1		1	
**Suffering from any kind of major sickness**	Yes	1.83 (1.425–2.372)	<0.001	1.77 (1.46–2.13)	<0.001
	No	1		1	
**Covid infected**	Yes	1.21 (0.991–1.49)	0.06	1.28 (1.11–1.47)	<0.001
	No	1		1	
**GPA in HSC**	GPA5	1.47 (1.238–1.760)	<0.001	1.49 (1.27–1.75)	<0.001
	Not GPA5	1		1	
**Suffering from any kind of mental problem**	Yes	1.18 (0.630–1.126)	0.24	1.3 (0.62–0.96)	0.02
	No	1		1	
**Confidence level for exam preparation**	Confident	0.62 (0.461–0.847)	<0.001	0.48 (0.38–0.61)	<0.001
	Less confident	0.51 (0.375–0.709)	<0.001	0.43 (0.33–0.55)	<0.001
	More confident	0.30 (0.210–0.439)	<0.001	0.19 (0.14–0.27)	<0.001
	Not confident	1		1	

## Discussion

The research was meant to find the prevalence of depressive symptoms among undergraduate admission candidates in Bangladesh, as well as to examine the influence of various socio-demographic factors, including the impacts of the Covid-19 pandemic, on these symptoms. The findings of our analyses indicated that a significant proportion, specifically 74%, of individuals who submitted applications for undergraduate study at universities reported experiencing symptoms indicative of depression. The survey findings also indicated a significant association between depression and 11 out of the 21 potential factors. The variables considered in this study encompassed gender, frequency of daily exercise, engagement in pre-marital affairs, number of daily study hours, religious practices, experiences of blackmail, family unrest, significant illnesses, COVID-19 infection status, GPA, presence of any mental health issues, and level of confidence for the admission test.

A a study conducted in China among admission seekers, revealed that 47% of individuals seeking admission experienced symptoms of depression [26]. Another research conducted in Bangladesh revealed that approximately 50% of students experience symptoms of depression [27]. The current investigation revealed a rate of 74%. This finding demonstrates a significant increase compared to previous research.

Several studies found gender as a determinant of depression among adolescents, as well as admission seekers [28,29]. There is evidence to support the presence of gender-related subtypes of depression, among which the developmental subtype seems to have the most significant potential in contributing to the gender difference [30]. One of the significant findings of the study was that female students were found more depressed than male students.

Family has a great impact on the mental well-being of the human species [31]. Researchers concluded that conflict within the family and a strained connection with one’s parents were significantly connected to an increased incidence of depression in adolescents [32]. Participants of our study had family unrest, and we found it as one of the potential factors for increasing depression among the participants. Those who had family problems were about 3 times more prone to depression.

The global pandemic resulting from the Covid-19 virus has had a significant impact on the physical and emotional well-being of individuals worldwide. Adolescents suffered from several mental health disorders including depression, anxiety, and even suicide [33]. The narrative assessment of research articles yielded the finding that the mental well-being of adolescents was adversely affected because of the preventive measures implemented during the pandemic [34]. The participants of our study also greatly suffered from the covid-19, and the infected students were more depressed than the students who were not affected by the virus.

According to research, exercise had not appeared to prevent adolescents from developing depressive symptoms [35]. Other researchers, however, found a link between depression and physical exercise [36,37]. The results of the present research provided empirical evidence in support of the notion that a significant association exists between physical exercise and depression. The findings of the study indicated that those who engaged in daily exercise had lower levels of depression compared to individuals who did not engage in regular exercise.

There is growing evidence that frequent use of social media may have a negative influence on users’ mental health, particularly among younger generations. A systematic review of 13 studies concluded that frequent use of social media by adolescents was associated with a higher likelihood of developing mental health disorders such as low self-esteem, stress, anxiety, and depression [38]. Even though the use of social media as a variable was not found to be significant in our ordinal logistic model, Pearson Chi-squared test identified a significant association between social media use and depression.

The result of the previous academic examination (GPA) is also an important factor for the undergraduate entrance exam [39]. This study exhibits GPA as a significant factor in depression. Higher GPA boosts the confidence level of admission candidates, and thus it helped to reduce depression and lower GPA candidates had a higher rate of depression. A previous study also found GPA as a promising factor for entrance test participants and adolescents [40].

This study’s findings implied that study time influences depression. The length of the study boosts candidates’ confidence. According to a study, confidence levels reduced depression [41]. The research undertaken on the entry participants yielded the same results [42]. Those who spend more time studying, on the other hand, have a higher level of confidence. Those who studied less exhibited lower levels of confidence. And, as indicated by another study [43], this was directly related to depression among the candidates.

One study looked at latent religiosity in nonreligious thirteen to fifteen-year-olds in England and Wales, considering attachment to Christian passage rituals as one example of implicit religion. The study showed that young people who adhere to implicit religion have greater psychological health [44]. The participants in this study expressed a strong desire to practice religion which reduced their depression. Those who practiced religion had less depression than those who did not practice religion so well.

Researchers mentioned pre-martial relationships as one of the key factors of adolescent depression [45]. It also increased suicidal behavior among adolescents [46]. This research corroborated the previously established observation that there is a greater prevalence of depression among married teenagers who had engaged in pre-marital relationships. One potential explanation might be attributed to the prevailing social and cultural norms surrounding arranged marriages.

The psychological well-being of adolescents was also significantly affected when they are the targets of blackmailing [47]. Researchers pointed out a direct link between blackmailing and the rise in the level of depression. This study also assessed blackmailing as a potential factor that contributes to depression among the participants to increase. The act of engaging in blackmailing or any kind of victimization might result in increased susceptibility to abnormal stress sensitivity among victims, hence potentially exacerbating mental health issues such as depression and anxiety [48].

Though some studies defined smoking, age, living area, and family income as enhancers of depression [49–52]; this research had not inaugurated any significant interaction with these factors. In the present study, there was no significant relation between age, smoking, living area, economic condition of family, and depression among the undergrad admission candidates of Bangladesh.

### Limitations

The key strength of this research was its sample size. It was conducted on primary data with a large sample size (over 5000 responses) that considers various socio-demographic variables, variables regarding academic conditions, personal habits, as well as mental health measurements, providing new perspectives on potential factors of depression among stressed-out students. The vast majority of the nation’s population may be accounted for as responders were taken from a variety of areas. In certain instances, an online questionnaire may result in a misunderstanding of the question, leaving the replies susceptible to lack of integrity. The interpretation of findings from an ordinal logistic model is very complex and difficult to grasp. The cross-sectional design of this study was also a limitation. Number of 2nd time examinees was substantially less in number than that of 1st time, hence insufficient to compare as a factor of depression. It is anticipated that this topic will be addressed in future studies. Notwithstanding these limitations, this research presents, for the first instance, comprehensive depression benchmarking outcomes at the group level.

### Conclusion

Our analysis reveals that many Bangladeshi undergraduate admission candidates have clinical depression or are at risk for it. The level of confidence in being admitted, the length of their study period, the possibility of blackmail, pre-marital relationship, their result, family unrest, and religiosity all affect their mental health. Stakeholders (such as family and teachers) need to work together to provide mental health care programs to lessen the severity of the issue. To reduce the burden on their minds and overcome depression, students should learn to deal with their mental stabilities. They should increase religiosity and physical exercise and try to avoid the complexities of pre-marital relationships, and if anyone is facing any kind of blackmail, they should talk with the valid authorities.

## Supporting information

S1 Appendix (DOCX)

## References

[1] Auerbach RP, Alonso J, Axinn WG, Cuijpers P, Ebert DD, Green JG, et al. Mental disorders among college students in the World Health Organization World Mental Health Surveys. Psychol Med. 2016 Oct 1;46(14):2955–70. 27484622 10.1017/S0033291716001665 PMC5129654

[2] Faye-Dumanget C, Carré J, Le Borgne M, Boudoukha PAH. French validation of the Maslach Burnout Inventory-Student Survey (MBI-SS). J Eval Clin Pract. 2017 Dec 1;23(6):1247–51. 28653800 10.1111/jep.12771

[3] Mamun MA, Misti JM, Hosen I, al Mamun F. Suicidal behaviors and university entrance test-related factors: A Bangladeshi exploratory study. Perspect Psychiatr Care. 2022 Jan 1;58(1):278–87. 33834493 10.1111/ppc.12783

[4] Tribune D. Over 95% of students clear HSC, equivalent exams | Dhaka Tribune [Internet]. 2022 [cited 2023 Feb 3]. Available from: https://www.dhakatribune.com/education/2022/02/13/over-95-of-students-clear-hsc-equivalent-exams.

[5] BANBEIS. Summary Statistics and Key Performance Indicators (KPI) A: Summary 1. In.

[6] Protikuzzaman M, Baowaly MK, Devnath MK, Singh BC. Predicting undergraduate admission: a case study in Bangabandhu Sheikh Mujibur Rahman Science and Technology University, Bangladesh. International Journal of Advanced Computer Science and Applications. 2020;11(12):138–45.

[7] Tribune D. Student commits suicide over failure to pass medical college admission test in Dhaka | Dhaka Tribune [Internet]. Dhaka Tribune. 2021 [cited 2023 Feb 3]. Available from: https://archive.dhakatribune.com/bangladesh/2021/04/07/student-commits-suicide-over-failure-to-pass-medical-college-admission-test-in-dhaka.

[8] Rojas M. 19 Students Commit Suicide After Failing their College Admissions Test–Anaheim Exclusivo [Internet]. ae news. 2019 [cited 2023 Feb 3]. Available from: https://aenews.org/2940/in-our-world/19-students-commit-suicide-after-failing-their-college-admissions-test/.

[9] Jha KK, Singh SK, Nirala SK, Kumar C, Kumar P, Aggrawal N. Prevalence of depression among school-going adolescents in an Urban Area of Bihar, India. Indian J Psychol Med. 2017;39(3):287–92. doi: 10.4103/0253-7176.207326 28615762 PMC5461838

[10] Roohafza H, Omidi R, Alinia T, Heidari K, Mohammad-Shafiee G, Jaberifar M, et al. Factors associated with smoking contemplation and maintenance among iranian adolescents. Eastern Mediterranean Health Journal. 2018;24(8):714–21. doi: 10.26719/2018.24.8.714 30328601

[11] Khalid A, Qadir F, Chan SWY, Schwannauer M. Adolescents’ mental health and well-being in developing countries: a cross-sectional survey from Pakistan. Journal of Mental Health. 2019;28(4):389–96. doi: 10.1080/09638237.2018.1521919 30451053

[12] Ang AL, Wahab S, Abd Rahman FN, Hazmi H, Md Yusoff R. Depressive symptoms in adolescents in Kuching, Malaysia: Prevalence and associated factors. Pediatrics International. 2019;61(4):404–10. doi: 10.1111/ped.13778 30597707

[13] Rodrigo C, Welgama S, Gurusinghe J, Wijeratne T, Jayananda G, Rajapakse S. Symptoms of anxiety and depression in adolescent students; a perspective from Sri Lanka. Child Adolesc Psychiatry Ment Health. 2010;4:10–2. doi: 10.1186/1753-2000-4-10 20334654 PMC2855518

[14] Hesketh T, Ding QJ. Anxiety and depression in adolescents in urban and rural China. Psychol Rep. 2005 Apr;96(2):435–44. doi: 10.2466/pr0.96.2.435-444 15941121

[15] CHO SJ, JEON HJ, KIM MJ, KIM JK, KIM US, LYOO IK, et al. Prevalence and Correlates of Depressive Symptoms among the Adolescents in an Urban Area in Korea. Journal of Korean Neuropsychiatric Association. 2001;627–39.

[16] Sayed M, Naiim CM, Aboelsaad M, Ibrahim MK. Internet addiction and relationships with depression, anxiety, stress and academic performance among Egypt pharmacy students: a cross-sectional designed study. BMC Public Health. 2022 Dec 1;22(1):1–10. 36163012 10.1186/s12889-022-14140-6 PMC9513952

[17] Ribeiro JD, Franklin JC, Fox KR, Bentley KH, Kleiman EM, Chang BP, et al. Self-injurious thoughts and behaviors as risk factors for future suicide ideation, attempts, and death: a meta-analysis of longitudinal studies. Psychol Med. 2016 Jan 1;46(2):225–36. doi: 10.1017/S0033291715001804 26370729 PMC4774896

[18] Wilkinson AL, Halpern CT, Herring AH. Directions of the relationship between substance use and depressive symptoms from adolescence to young adulthood. Addictive Behaviors. 2016 Sep 1;60:64–70. doi: 10.1016/j.addbeh.2016.03.036 27100470 PMC4884464

[19] Chen F, Chen J, Chen B, Mofatteh M, Wen C, Wellington J, et al. Mental health status of medical students during postgraduate entrance examination. BMC Psychiatry. 2022 Dec 1;22(1):1–10. 36575395 10.1186/s12888-022-04482-1 PMC9793374

[20] Karatas H, Alci B, Aydin H. Correlation among high school senior students’ test anxiety, academic performance and points of university entrance exam. Educational Research and Reviews. 2013;8(13):919–26.

[21] Kavakci O, Semiz M, Kartal A, Dikici A, Kugu N. Test anxiety prevalance and related variables in the students who are going to take the university entrance examination. The Journal of Psychiatry and Neurological Sciences. 2014;27:301–7.

[22] Repon MAU, Pakhe SA, Quaiyum S, Das R, Daria S, Islam MR. Effect of COVID-19 pandemic on mental health among Bangladeshi healthcare professionals: A cross-sectional study. Sci Prog. 2021 Jun 24;104(2). doi: 10.1177/00368504211026409 34166132 PMC10455000

[23] Afrin S, Nasrullah SM, Dalal K, Tasnim Z, Benzadid MS, Humayra F, et al. Mental health status of adolescents in-home quarantine: a multi-region, cross-sectional study during COVID-19 pandemic in Bangladesh. BMC Psychol. 2022 Dec 1;10(1):1–12. 35513856 10.1186/s40359-022-00819-3 PMC9069420

[24] Nafiu RF. Admission test of 20 public universities postponed. Dhaka Tribune. 2020.

[25] Kroenke K, Spitzer RL, Williams JBW, Löwe B. The Patient Health Questionnaire Somatic, Anxiety, and Depressive Symptom Scales: a systematic review. Gen Hosp Psychiatry. 2010 Jul;32(4):345–59. doi: 10.1016/j.genhosppsych.2010.03.006 20633738

[26] Yang W, Sun R, Wang C, Chen J, Zhang C, Yu J, et al. Epidemiology of depressive disorders among youth during Gaokao to college in China: results from Hunan Normal University mental health survey. BMC Psychiatry 2023 23:1. 2023 Jun 29;23(1):1–18. 37386434 10.1186/s12888-023-04972-w PMC10308668

[27] Sakib N, Islam M, Al Habib MS, Bhuiyan AKMI, Alam MM, Tasneem N, et al. Depression and suicidality among Bangladeshi students: Subject selection reasons and learning environment as potential risk factors. Perspect Psychiatr Care. 2021 Jul 1;57(3):1150–62. doi: 10.1111/ppc.12670 33135191

[28] Salk RH, Hyde JS, Abramson LY. Gender differences in depression in representative national samples: Meta-analyses of diagnoses and symptoms. Psychol Bull. 2017 Aug 1;143(8):783–822. doi: 10.1037/bul0000102 28447828 PMC5532074

[29] Mamun MA, Misti JM, Hosen I, al Mamun F. Suicidal behaviors and university entrance test-related factors: A Bangladeshi exploratory study. Perspect Psychiatr Care. 2022 Jan 1;58(1):278–87. doi: 10.1111/ppc.12783 33834493

[30] Kuehner C. Why is depression more common among women than among men? Lancet Psychiatry. 2017 Feb 1;4(2):146–58. doi: 10.1016/S2215-0366(16)30263-2 27856392

[31] Koerner AF, Schrodt P. An Introduction to the Special Issue on Family Communication Patterns Theory. https://doi.org/101080/152674312013857328. 2014 Jan;14(1):1–15.

[32] Consoli A, Peyre H, Speranza M, Hassler C, Falissard B, Touchette E, et al. Suicidal behaviors in depressed adolescents: Role of perceived relationships in the family. Child Adolesc Psychiatry Ment Health. 2013 Mar 16;7(1):1–12. 23497551 10.1186/1753-2000-7-8 PMC3655930

[33] Fegert JM, Vitiello B, Plener PL, Clemens V. Challenges and burden of the Coronavirus 2019 (COVID-19) pandemic for child and adolescent mental health: A narrative review to highlight clinical and research needs in the acute phase and the long return to normality. Child Adolesc Psychiatry Ment Health. 2020 May 12;14(1):1–11. 32419840 10.1186/s13034-020-00329-3 PMC7216870

[34] Singh S, Roy D, Sinha K, Parveen S, Sharma G, Joshi G. Impact of COVID-19 and lockdown on mental health of children and adolescents: A narrative review with recommendations. Psychiatry Res. 2020 Nov 1;293:113429. doi: 10.1016/j.psychres.2020.113429 32882598 PMC7444649

[35] Toseeb U, Brage S, Corder K, Dunn VJ, Jones PB, Owens M, et al. Exercise and depressive symptoms in adolescents: A longitudinal cohort study. JAMA Pediatr. 2014 Dec 1;168(12):1093–100. doi: 10.1001/jamapediatrics.2014.1794 25317674

[36] Naderi S, Naderi S, Delavar A, Dortaj F. The effect of physical exercise on anxiety among the victims of child abuse. Sport Sci Health. 2019 Dec 1;15(3):519–25.

[37] Field T. Exercise research on children and adolescents. Vol. 18, Complementary Therapies in Clinical Practice. Churchill Livingstone; 2012. p. 54–9. doi: 10.1016/j.ctcp.2011.04.002 22196575

[38] Keles B, McCrae N, Grealish A. A systematic review: the influence of social media on depression, anxiety and psychological distress in adolescents. Int J Adolesc Youth. 2019 Jan 2;25(1):79–93.

[39] Rolim LA, Santos FCM dos, Chaves LL, Gonçalves MLCM, Freitas-Neto JL, Nascimento AL da S do, et al. The validity of University Entrance Examination and High school Grade point average for predicting first year university students’academic performance. Nature. 2020;388:539–47.

[40] Hysenbegasi A, Hass SL, Rowland CR. The impact of depression on the academic productivity of university students. Journal of Mental Health Policy and Economics. 2005;8(3):145–51. 16278502

[41] Koyama A, Matsushita M, Ushijima H, Jono T, Ikeda M. Association between depression, examination-related stressors, and sense of coherence: The ronin-sei study. Psychiatry Clin Neurosci. 2014 Jun 1;68(6):441–7. doi: 10.1111/pcn.12146 24506541

[42] Mamun MA, Misti JM, Hosen I, al Mamun F. Suicidal behaviors and university entrance test-related factors: A Bangladeshi exploratory study. Perspect Psychiatr Care. 2022;58(1):278–87. doi: 10.1111/ppc.12783 33834493

[43] Kavakci O, Semiz M, Kartal A, Dikici A, Kugu N. Test anxiety prevalance and related variables in the students who are going to take the university entrance examination. Dusunen Adam. 2014;27(4):301–7.

[44] Francis LJ, Penny G. Implicit religion and psychological wellbeing: A study among adolescents without formal religious affiliation or practice. Implicit Religion. 2016;19(1):61–78.

[45] Roberts TA, Klein J. Intimate partner abuse and high-risk behavior in adolescents. Arch Pediatr Adolesc Med. 2003 Apr 1;157(4):375–80. doi: 10.1001/archpedi.157.4.375 12695234

[46] Roberts TA, Klein JD, Fisher S. Longitudinal effect of intimate partner abuse on high-risk behavior among adolescents. Vol. 157, Archives of Pediatrics and Adolescent Medicine. Arch Pediatr Adolesc Med; 2003. p. 875–81. doi: 10.1001/archpedi.157.9.875 12963592

[47] Medrano JLJ, Lopez Rosales F, Gámez-Guadix M. Assessing the Links of Sexting, Cybervictimization, Depression, and Suicidal Ideation Among University Students. Archives of Suicide Research. 2018 Jan 2;22(1):153–64. doi: 10.1080/13811118.2017.1304304 28287925

[48] Ren P, Liu B, Xiong X, Chen J, Luo F. The longitudinal relationship between bullying victimization and depressive symptoms for middle school students: A cross-lagged panel network analysis. J Affect Disord. 2023 Aug 10; 10.1016/j.jad.2023.08.048 37572700

[49] Kenney BA, Holahan CJ. Depressive symptoms and cigarette smoking in a college sample. Journal of American College Health. 2008;56(4):409–14. doi: 10.3200/JACH.56.44.409-414 18316285

[50] Bhandari M. Anxiety and depression among adolescent students at higher secondary school. BIBECHANA. 2016 Nov 28;14:103–9.

[51] Tjora T, Hetland J, Aarø LE, Wold B, Wiium N, Øverland S. The association between smoking and depression from adolescence to adulthood. Addiction. 2014 Jun 1;109(6):1022–30. doi: 10.1111/add.12522 24552489

[52] Uecker JE. Marriage and Mental Health among Young Adults. J Health Soc Behav. 2012 Feb 9;53(1):67–83. doi: 10.1177/0022146511419206 22328171 PMC3390929

