# Supplementary material for: Prevalence of depression and its associated factors among undergraduate admission candidates in Bangladesh: A nation-wide cross-sectional study
Source: PLoS One. 2023 Nov 30;18(11):e0295143. doi: 10.1371/journal.pone.0295143 (PMC10688886; doi:10.1371/journal.pone.0295143)
Supplement: S1 Appendix — (DOCX) [file pone.0295143.s001.docx]

| Appendix 1 | |
| --- | --- |
| Number | PHQ Questions |
| 01 | In doing things, there is little interest or pleasure |
| 02 | Hopeless or depressed, feeling down, |
| 03 | Sleeping too much or staying asleep or having trouble falling |
| 04 | Having little energy or feeling tired |
| 05 | Overeating or poor appetite |
| 06 | Feeling uncomfortable about yourself - or have let yourself or your family down or that you are a failure |
| 07 | Trouble concentrating on things, such as watching television or reading the newspaper |
| 08 | So slowly moving or speaking that can be noticed by other people |
| 09 | Better thoughts are hurting yourself or dead |
